# The carriage of interleukin-1B-31*C allele plus *Staphylococcus aureus* and *Haemophilus influenzae* increases the risk of recurrent tonsillitis in a Mexican population

**DOI:** 10.1371/journal.pone.0178115

**Published:** 2017-05-24

**Authors:** Baltazar González-Andrade, Ramiro Santos-Lartigue, Samantha Flores-Treviño, Natalie Sonia Ramirez-Ochoa, Paola Bocanegra-Ibarias, Francisco J. Huerta-Torres, Soraya Mendoza-Olazarán, Licet Villarreal-Treviño, Adrián Camacho-Ortiz, Hipólito Villarreal-Vázquez, Elvira Garza-González

**Affiliations:** 1 Servicio de Otorrinolaringología, Hospital Universitario “Dr. José Eleuterio González”, Universidad Autónoma de Nuevo León, Monterrey, Nuevo Leon, Mexico; 2 Servicio de Gastroenterología, Hospital Universitario “Dr. José Eleuterio González”, Universidad Autónoma de Nuevo León, Monterrey, Nuevo Leon, Mexico; 3 Hospital Materno Infantil de Alta Especialidad, Secretaria de Salud de Nuevo Leon, Monterrey, Nuevo Leon, Mexico; 4 Departamento de Microbiología, Facultad de Ciencias Biológicas, Universidad Autónoma de Nuevo León, Monterrey, Nuevo Leon, Mexico; 5 Coordinación de Epidemiología Hospitalaria, Hospital Universitario Dr. José Eleuterio González, Universidad Autónoma de Nuevo León, Monterrey, Nuevo León, Mexico; Public Health England, UNITED KINGDOM

## Abstract

The aim of the present study was to estimate the relative contribution of immunogenetic and microbiological factors in the development of recurrent tonsillitis in a Mexican population. Patients (n = 138) with recurrent tonsillitis and an indication of tonsillectomy (mean age: 6.05 years ± 3.00; median age: 5 years, female: 58; age range: 1–15 years) and 195 non-related controls older than 18 years and a medical history free of recurrent tonsillitis were included. To evaluate the microbial contribution, tonsil swab samples from both groups and extracted tonsil samples from cases were cultured. Biofilm production of isolated bacteria was measured. To assess the immunogenetic component, DNA from peripheral blood was genotyped for the *TNFA-308G/A* single-nucleotide polymorphism (SNP) and for the *IL1B -31C/T* SNP. Normal microbiota, but no pathogens or potential pathogens, were identified from all control sample cultures. The most frequent pathogenic species detected in tonsils from cases were *Staphylococcus aureus* (48.6%, 67/138) and *Haemophilus influenzae* (31.9%, 44/138), which were found more frequently in patient samples than in samples from healthy volunteers (*P* < 0.0001). Importantly, 41/54 (75.9%) *S*. *aureus* isolates were biofilm producers (18 weak and 23 strong), whereas 17/25 (68%) *H*. *influenzae* isolates were biofilm producers (10 weak, and 7 strong biofilm producers). Patients with at least one copy of the IL1B-31*C allele had a higher risk of recurrent tonsillitis (OR = 4.03; 95% CI = 1.27–14.27; *P* = 0.013). *TNFA-308 G/A* alleles were not preferentially distributed among the groups. When considering the presence of IL1B-31*C plus *S*. *aureus*, IL1B-31*C plus *S*. *aureus* biofilm producer, IL1B-31*C plus *H*. *influenzae* or IL1B-31*C plus *H*. *influenzae* biofilm producer, the OR tended to infinite. Thus, the presence of IL1B-31*C allele plus the presence of *S*. *aureus* and/or *H*. *influenzae* could be related to the development of tonsillitis in this particular Mexican population.

## Introduction

Recurrent tonsillitis is a childhood disease that involves the parenchyma of the palatine tonsils. The definition of recurrent disease may vary, but the criteria used at present are the development of 5 or more episodes of true tonsillitis per year, symptoms recurring for at least a year, and episodes that are disabling and that prevent normal functioning [1].

Most of the upper respiratory tract diseases in childhood tend to improve with time, although recurrent tonsillitis does not resolve spontaneously and requires treatment, with the highest risk of presentation in patients younger than 15 years. Options available for the treatment of recurrent disease are surgical and medical care with equal outcomes [2].

Recurrent tonsillitis has been linked to a wide variety of infectious agents [3], with the most common being *Streptococcus pyogenes*, followed by *Staphylococcus aureus* and *Haemophilus influenzae* [4–6].

In the development of recurrent tonsillitis, the bacterial factor plays an important role, but it may not be the only one, because certain genetic backgrounds may be related to stronger inflammation. Genetic variants of cytokines may alter the inflammatory response, and several genes encoding pro-inflammatory cytokines.

Among the well-studied single-nucleotide polymorphisms (SNPs) that may affect the inflammatory response is *TNFA-308G/A* (rs1800629), with guanine (G) as the common variant and adenine (A) as the less common one. The *TNFA–308A* allele shows a 6-to-7-fold higher gene transcription as compared to the common G allele [7, 8].

Furthermore, the –*31C/T* SNP (rs1143627) in the promoter of the *IL1B* gene has been associated with stronger inflammation in multiple populations, including the Mexican [9–11].

Genetic polymorphisms have been reported as risk factors for the development of recurrent tonsillitis, but the evidence is not strong enough [1]. For example, a case-control study indicated that parental atopy and a parental history of tonsillectomy would predict subsequent tonsillitis in their children [12].

The aim of the present study was to estimate the relative contribution of immunogenetic and microbiological factors in the development of recurrent tonsillitis in a Mexican population.

## Methods

### Study population

We included 138 unrelated patients with clinically confirmed recurrent tonsillitis and an indication of tonsillectomy according to American Academy of Otolaryngology–Head and Neck Surgery [13, 14] (mean age, 6.05 years ± SD, 3.00; median age, 5 years, Female: 58: age range: 1–15 years). We also investigated 195 healthy subjects with no clinical history of recurrent tonsillitis (mean age = 28.18 years ± SD, 10.26, Female: 91; median age = 23 years, age range: 18–68 years) as controls. All controls were older than 18 years to minimize the risk of recurrent tonsillitis. The recruitment time was from April, 2015 to April, 2016.

Patients were enrolled from August, 2014 to May, 2015 at two hospitals from Nuevo Leon, Mexico. This study was performed with the approval of the Local Ethics Committee of both hospitals: Hospital Universitario “Dr. José Eleuterio González”, Universidad Autónoma de Nuevo León (Approval OTI-007) and Hospital Materno Infantil de Alta Especialidad, Secretaria de Salud de Nuevo Leon (Approval 062/2014). Written informed consent was obtained from all subjects and patients. When applied, written informed consent was obtained from caretakers, or guardians on behalf of the minors enrolled in this study.

### Microbiological examinations and semi-quantification of biofilm

From all control subjects, a tonsil swab culture was obtained for microbiological evaluation. From patients with recurrent tonsillitis, extracted tonsils (partially macerated) and tonsil swabs were cultured by standard methods.

Biofilm production by *S*. *aureus* and *H*. *influenzae* isolates was semi-quantified spectrophotometrically at an optical density of 595 nm (OD_595_: OD_595_ < 0.12, non-biofilm producers; 0.13<OD_595_ < 0.24, weak biofilm producers; and OD_595_ > 0.25, strong biofilm producers) after crystal violet staining according to methods previously reported either for *S*. *aureus* [15] or for *H*. *influenzae* [16, 17]. Control strains were *S*. *aureus* (ATCC 29213; strong biofilm producer), *Escherichia coli (*ATCC 25922, weak biofilm producer), and *Staphylococcus hominis* (ATCC 27844; no biofilm producer). All isolates were tested in quadruplicate in two different experiments carried out on different days.

### Genotyping

Genomic DNA was extracted from peripheral blood from both control and subjects by the phenol-chloroform-isoamyl alcohol method and precipitated with ethanol.

The *IL1B -31T/C* SNP (rs1143627) was evaluated by restriction fragment length polymorphism (PCR-RFLP) and the *TNFA-308G/A* SNP (rs1800629) by pyrosequencing as previously described [18].

### Statistical analysis

The Hardy-Weinberg equilibrium of alleles was assessed with a chi-square test. Statistically significant differences were determined by 2-tailed Student's *t*, chi-square, or Fisher’s exact tests. A *P* value of < 0.05 was considered significant. Odds ratio (OR) and corresponding 95% confidence interval (95% CIs) were computed using SPSS Statistics software version 20.0 (IMB Corporation). The dominant model of inheritance was used for analysis.

## Results

### Microbiological results

Normal microbiota, but no potential pathogens, grew in cultures from control samples.

Considering cultures from both swab and extracted tonsil samples, *S*. *aureus* and *H*. *influenzae* were found more frequently in patients with recurrent tonsillitis than in control subjects (*P*<0.0001). Cultures from extracted tonsils presented a higher diversity and quantity of potential pathogens; the most frequently detected pathogens were *S*. *aureus* (67/138; 48.6%), *H*. *influenzae* (44/138; 31.9%) and *S*. *pyogenes* 21/138 (15.2%) (Table 1).

**Table 1:** Microbiological evaluation from extracted tonsils and swab tonsils from all cases.

No. of microorganisms isolated	Tonsils swab n = 138 n(%) *	Extracted tonsils n = 138 n(%)
1	65 (47.1)	71 (51.4)
2	26 (18.8)	36 (26.1)
3	6 (4.3)	11 (8.0)
**Microorganism isolated**		
*Staphylococcus aureus*	53 (38.4)	67 (48.6)
*Haemophilus influenzae*	30 (21.7)	44 (31.9)
*Streptococcus pyogenes*	15 (10.9)	21 (15.2)
*Pseudomonas aeruginosa*	10 (7.2)	10 (7.2)
*Enterobacter cloacae*	4 (2.9)	5 (3.6)
*Streptococcus agalactiae*	2 (1.4)	5 (3.6)
*Citrobacter freundii*	6 (4.3)	4 (2.9)
*Klebsiella pneumoniae*	4 (2.9)	4 (2.9)
Group G β-hemolytic *Streptococcus*	3 (2.2)	4 (2.9)
*Streptococcus pneumoniae*	1 (0.7)	3 (2.2)
*Candida* spp.	2 (1.4)	2 (1.4)
*Klebsiella oxytoca*	2 (1.4)	1 (0.7)
*Pantoea agglomerans*	2 (1.4)	1 (0.7)
*Burkholderia cepacia*	1 (0.7)	1 (0.7)
*Acinetobacter baumannii*	0 (0)	1 (0.7)
*Serratia plymuthica*	0 (0)	1 (0.7)
*Pseudomonas pseudoalcaligenes*	0 (0)	1 (0.7)
*Chryseobacterium indologenes*	0 (0)	1 (0.7)

* All control subjects (n = 195) exhibited normal microbiota in the tonsils swab samples. Extracted tonsils were analyzed only in case patients.

We randomly selected 54 *S*. *aureus* isolates for biofilm evaluation and detected that 41/54 (75.9%) were biofilm producers (18 weak and 23 strong). Among *H*. *influenzae* isolates, 25 isolates were randomly selected for biofilm evaluation and 17/25 (68%) were biofilm producers (10 weak and 7 strong biofilm producers).

### Allelic and genotypic associations

The distribution of alleles and genotypes is shown in Table 2. The genotype frequencies were in Hardy-Weinberg equilibrium for all loci studied. Patients with at least one copy of the IL1B-31*C allele had a higher risk of recurrent tonsillitis (OR = 4.03; 95% CI = 1.27–14.27; *P* = 0.013). There were no significant differences for the *TNFA-308* locus.

**Table 2:** Genotype and allele frequencies of *TNFA-308G/A* and *IL1B-31C/T* in recurrent tonsillitis and control patients.

	Genotype frequency n (%)			Allele frequency		Odds ratio (95% CI)	*P*
***IL1B-31C/T***	CC	CT	TT	**C**	**T**		
Cases (n = 138)	56 (40.6)	78 (56.5)	4 (2.9)	0.69	0.31	4.03 (1.27–14.27)	0.013
Controls (n = 195)	81 (42.6)	93 (48.9)	21 (11.1)	0.65	0.35		
***TNFA-308G/A***	AA	GA	GG	**A**	**G**		
Cases (n = 138)	0 (0.0)	21(15.2)	117 (84.8)	0.08	0.92		NS
Controls (n = 195)	2 (1.1)	54 (28.4)	139 (73.2)	0.15	0.85		

NS: not significant.

Considering IL1B-31*C mutant allele in the control group, the power of our analysis (α = 0.05) was 0. 0.9853 in 138 cases and 195 controls.

When we considered the presence of IL1B-31*C plus *S*. aureus; IL1B-31*C plus *S*. *aureus* biofilm producer; IL1B-31*C plus *H*. influenzae IL1B-31*C plus *H*. *influenzae* biofilm producer, the OR tended to infinite (Table 3).

**Table 3:** Odds ratios (OR) with 95% confidence intervals (CIs) for risk factors.

	IL1B-31 *C	IL1B-31 *C + *S*. *aureus*	IL1B-31 *C + *S*. *aureus* biofilm producer	IL1B-31*C + *H*. *influenzae*	IL1B-31 *C + *H*. *influenzae* biofilm producer
Cases (n = 138)	134	67/134	38/52	34/134	15/25
Controls (n = 195)	174	0/174	0	0	0
OR (95%CI), *P*	4.03(1.27–14.27), 0.013	∞ (39.21-∞), 0.0	∞(14.436-∞), 0.0	∞ (12.387-∞), 0.0	∞ (4.46-∞), 0.0

## Discussion

In this study, we aimed to analyze the immunogenetic and microbiological contributions to the development of recurrent tonsillitis. Though cultures from either a swab or extracted tonsil sample from the same patient were consistent in about 60% of the cases, in the remaining 40% the cultures from extracted tonsils tended to have higher yields, both in number and species diversity, including pathogenic species.

Intracellular *S*. *aureus* has been reported as the most common cause of recurrent tonsillitis, and it has been suggested that *S*. *aureus* uses the intracellular location to survive the effects of antibiotics and the host immune response [19], which could explain the discrepancy between extracted tonsil and swab cultures in some cases, as infected cells at deeper tissue layers would be missed in swab cultures of superficial cells. Interestingly, cultures from control samples were limited to normal microbiota and no potential pathogen was detected, which could be explained by the inclusion criteria of the control group: 1) the absence of recurrent tonsillitis in their medical history and 2) their age; over 18 years is beyond the risk period of recurrent tonsillitis). Additionally, the sampling method was limited to superficial cells only which does not rule out that the control subjects could have any of the potential intracellular pathogens or crypt depth that could not be detected by the superficial culture of tonsils. Of course, ethical considerations do not impair the biopsy of the controls.

In our study cases were all from pediatrics patients (median age, 5 years) while controls were > 18 years (median age, 28 years).We included older controls to minimize the immunogenetic risk for future recurrent tonsillitis. In case-control studies, the only variables should be the ones under study and differential work-up between control and case groups should be avoided. However, due to ethical reasons, biopsies were not obtained from controls. Culture from extracted tonsil facilitated the detection of more bacterial species, including potentially pathogenic ones. If we considered only the swab results when comparing patients with controls, the percentages of potential pathogens detected are lower, but equally significant. Overall, the most frequent pathogenic species were *S*. *aureus* and *H*. *influenzae*, which is consistent with the literature [19, 20].

The impact of biofilm formation has been previously reported in a case-control study that included 20 patients with recurrent tonsillitis and another 20 with no history of tonsillitis in the previous 2 years. Biofilm prevalence, analyzed by scanning electron microscopy, was significantly more prevalent in the recurrent tonsillitis group than in the control group [21]. Similarly, another study, in which *H*. *influenzae* was the most frequent pathogen in adenotonsillitis patients, proposed that biofilms could have an aetiopathogenetic role in chronic inflammatory mucosal reactions [20]. In this scenario, the presence of *H*. *influenzae* and *S*. *aureus*, especially in biofilms, may have a role in the development of the tonsillar inflammatory disease and an inadequate pro-inflammatory genetic background may increase this role.

The genetically regulated immune response seems to play a crucial role in determining the intensity of damage to the tonsils [22]. We found an association between the presence of the IL1B-31*C allele (both homozygote plus heterozygote) and the development of recurrent tonsillitis. When we added the bacterial factor (*S*. *aureus* and *H*. *influenza*e) plus the production of biofilm, the OR increased to infinite.

Given these results, we hypothesize that a genetically conditioned inflammatory response (by the presence of IL1B-31*C allele) and the infection with *S*. *aureus* and/or *H*. *influenzae* may lead to recurrent tonsillitis. To our knowledge, so far no other study has shown this association.

One of the few studies that have analyzed the genetic immune response and the development of recurrent tonsillitis showed that the presence of the Tool-Like receptor (TLR) 4-T399I polymorphism was associated with a 2-fold decreased risk of *H*. *influenzae* carriage (OR = 0.38, 95% CI = 0.15 to 0.96, P = 0.038); underlying that the host immune genetic variations might play a significant role in the susceptibility to common infections and tonsillar diseases [23]. The main limitation of this study is that we included only culture for aerobic bacterial species and no anaerobic culture was performed; and some anaerobic species may play a significant role in recurrent tonsillitis.

In conclusion, our results suggest that the presence of IL1B -31*C allele may be a tonsillitis-enhancing factor, especially when species such as *S*. *aureus* or *H*. *influenzae* are present. If this effect is confirmed in populations other than the Mexican one, it may be used to detect patients at higher risk for recurrent tonsillitis.

## Supporting information

S1 File (XLS)

## References

[1] McKerrow WS. Recurrent tonsillitis. Am Fam Physician. 2002;66(9):1735–6. . 12449272

[2] Prim MP, de Diego JI, Larrauri M, Diaz C, Sastre N, Gavilan J. Spontaneous resolution of recurrent tonsillitis in pediatric patients on the surgical waiting list. Int J Pediatr Otorhinolaryngol. 2002;65(1):35–8. . 12127220 10.1016/s0165-5876(02)00128-3

[3] Bisno AL. Are cephalosporins superior to penicillin for treatment of acute streptococcal pharyngitis? Clin Infect Dis. 2004;38(11):1535–7. 10.1086/392520 . 15156438

[4] McIsaac WJ, Goel V, To T, Low DE. The validity of a sore throat score in family practice. CMAJ. 2000;163(7):811–5. ; 11033707 PMC80502

[5] Group ESTG, Pelucchi C, Grigoryan L, Galeone C, Esposito S, Huovinen P, et al Guideline for the management of acute sore throat. Clin Microbiol Infect. 2012;18 Suppl 1:1–28. 10.1111/j.1469-0691.2012.03766.x . 22432746

[6] Glezen WP, Clyde WA Jr., Senior RJ, Sheaffer CI, Denny FW. Group A streptococci, mycoplasmas, and viruses associated with acute pharyngitis. JAMA. 1967;202(6):455–60. . 4293014

[7] Wilson AG, Symons JA, McDowell TL, McDevitt HO, Duff GW. Effects of a polymorphism in the human tumor necrosis factor alpha promoter on transcriptional activation. Proc Natl Acad Sci U S A. 1997;94(7):3195–9. ; 9096369 10.1073/pnas.94.7.3195 PMC20345

[8] Abdallah AN, Cucchi-Mouillot P, Biteau N, Cassaigne A, Haras D, Iron A. Analysis of the polymorphism of the tumour necrosis factor (TNF) gene and promoter and of circulating TNF-alpha levels in heart-transplant patients suffering or not suffering from severe rejection. Eur J Immunogenet. 1999;26(4):249–55. . 10457886 10.1046/j.1365-2370.1999.00128.x

[9] El-Omar EM, Carrington M, Chow WH, McColl KE, Bream JH, Young HA, et al Interleukin-1 polymorphisms associated with increased risk of gastric cancer. Nature. 2000;404(6776):398–402. 10.1038/35006081 . 10746728

[10] Furuta T, El-Omar EM, Xiao F, Shirai N, Takashima M, Sugimura H, et al Interleukin 1beta polymorphisms increase risk of hypochlorhydria and atrophic gastritis and reduce risk of duodenal ulcer recurrence in Japan. Gastroenterology. 2002;123(1):92–105. . 12105837 10.1053/gast.2002.34156

[11] Garza-González E, Bosques-Padilla FJ, El-Omar E, Hold G, Tijerina-Menchaca R, Maldonado-Garza HJ, et al Role of the polymorphic IL-1B, IL-1RN and TNF-A genes in distal gastric cancer in Mexico. Int J Cancer. 2005;114(2):237–41. 10.1002/ijc.20718 . 15540224

[12] Capper R, Canter RJ. Is the incidence of tonsillectomy influenced by the family medical or social history? Clin Otolaryngol Allied Sci. 2001;26(6):484–7. . 11843928 10.1046/j.1365-2273.2001.00508.x

[13] Randel A. AAO-HNS Guidelines for Tonsillectomy in Children and Adolescents. Am Fam Physician. 2011;84(5):566–73. . 21888309

[14] Baugh RF, Archer SM, Mitchell RB, Rosenfeld RM, Amin R, Burns JJ, et al Clinical practice guideline: tonsillectomy in children. Otolaryngol Head Neck Surg. 2011;144(1 Suppl):S1–30. 10.1177/0194599810389949 . 21493257

[15] Christensen GD, Simpson WA, Younger JJ, Baddour LM, Barrett FF, Melton DM, et al Adherence of coagulase-negative staphylococci to plastic tissue culture plates: a quantitative model for the adherence of staphylococci to medical devices. J Clin Microbiol. 1985;22(6):996–1006. Epub 1985/12/01. ; 3905855 10.1128/jcm.22.6.996-1006.1985 PMC271866

[16] Puig C, Marti S, Hermans PW, de Jonge MI, Ardanuy C, Linares J, et al Incorporation of phosphorylcholine into the lipooligosaccharide of nontypeable *Haemophilus influenzae* does not correlate with the level of biofilm formation in vitro. Infect Immun. 2014;82(4):1591–9. 10.1128/IAI.01445-13 ; 24452688 PMC3993405

[17] Mizrahi A, Cohen R, Varon E, Bonacorsi S, Bechet S, Poyart C, et al Non typable-*Haemophilus influenzae* biofilm formation and acute otitis media. BMC Infect Dis. 2014;14:400 10.1186/1471-2334-14-400 ; 25037572 PMC4223365

[18] Garza-Gonzalez E, Bosques-Padilla FJ, Mendoza-Ibarra SI, Flores-Gutierrez JP, Maldonado-Garza HJ, Perez-Perez GI. Assessment of the toll-like receptor 4 Asp299Gly, Thr399Ile and interleukin-8–251 polymorphisms in the risk for the development of distal gastric cancer. BMC Cancer. 2007;7:70 10.1186/1471-2407-7-70 ; 17462092 PMC1868033

[19] Zautner AE, Krause M, Stropahl G, Holtfreter S, Frickmann H, Maletzki C, et al Intracellular persisting *Staphylococcus aureus* is the major pathogen in recurrent tonsillitis. PLoS One. 2010;5(3):e9452 Epub 2010/03/01. 10.1371/journal.pone.0009452 ; 20209109 PMC2830486

[20] Galli J, Calò L, Ardito F, Imperiali M, Bassotti E, Fadda G, et al Biofilm formation by Haemophilus influenzae isolated from adeno-tonsil tissue samples, and its role in recurrent adenotonsillitis. Acta Otorhinolaryngol Ital. 2007;27(3):134–8. ; 17883191 PMC2640046

[21] Woo JH, Kim ST, Kang IG, Lee JH, Cha HE, Kim DY. Comparison of tonsillar biofilms between patients with recurrent tonsillitis and a control group. Acta Otolaryngol. 2012;132(10):1115–20. Epub 2012/06/12. 10.3109/00016489.2012.689859 . 22690674

[22] Todorović MM, Zvrko EZ. Immunoregulatory cytokines and chronic tonsillitis. Bosn J Basic Med Sci. 2013;13(4):230–6. ; 24289758 10.17305/bjbms.2013.2330 PMC4334011

[23] Liadaki K, Petinaki E, Skoulakis C, Tsirevelou P, Klapsa D, Germenis AE, et al Toll-like receptor 4 gene (TLR4), but not TLR2, polymorphisms modify the risk of tonsillar disease due to Streptococcus pyogenes and Haemophilus influenzae. Clin Vaccine Immunol. 2011;18(2):217–22. 10.1128/CVI.00460-10 ; 21159925 PMC3067360

