# Correction: The carriage of interleukin-1B-31*C allele plus *Staphylococcus aureus* and *Haemophilus influenzae* increases the risk of recurrent tonsillitis in a Mexican population

**DOI:** 10.1371/journal.pone.0186627

**Published:** 2017-10-12

**Authors:** Baltazar González-Andrade, Ramiro Santos-Lartigue, Samantha Flores-Treviño, Natalie Sofia Ramirez-Ochoa, Paola Bocanegra-Ibarias, Francisco J. Huerta-Torres, Soraya Mendoza-Olazarán, Licet Villarreal-Treviño, Adrián Camacho-Ortiz, Hipólito Villarreal-Vázquez, Elvira Garza-González

The fourth author’s name is incorrect. The correct name is: Natalie Sofia Ramirez-Ochoa.
